# Failure mechanism and bearing force of CFRP strengthened square hollow section under compressive load

**DOI:** 10.1038/s41598-024-59752-7

**Published:** 2024-04-23

**Authors:** Can Huang, Yan-hui Wei, Ke-jian Ma, Zhuo-qun Liu, Peng-gang Tian, Bing-zhen Zhao

**Affiliations:** 1 Research Center of Space Structures, Guizhou University, Guiyang, 550025 China; 2 Key Laboratory for Structural Engineering of Guizhou Province, Guizhou University, Guiyang, 550025 China; 3 Future City Innovation Technology Co., Ltd., Shaanxi Construction Engineering Holding Group, Xian, 710000 China

**Keywords:** CFRP plate, Square hollow section, Compressive load, Finite element model, Theoretical analysis, Engineering, Civil engineering

## Abstract

Carbon fibre-reinforced polymer (CFRP) plates can efficiently repair or enhance the mechanical properties of the square hollow section. However, the loading end of such a CFRP-strengthened member is prone to local bearing failure under compressive load. Given this limitation, an innovative CFRP-plate-strengthened square hollow section composite member (CFRP-SHSCM) was raised, and the thick-walled section was welded on both ends of the thin-walled steel column. The mechanical properties of CFRP-SHSCMs were investigated through parameter finite element (FE) analysis, focusing on the influence of the amount of CFRP layers (*n*_*c*_), the slenderness ratio (*λ*), the initial geometric imperfections (*v*_*0*_), the CFRP layouts (2S and 4S) and the length of the exposed steel column (*L*_*e*_). The load–displacement curves, the bearing force, and typical failure modes were also acquired. Results indicated that with increasing *n*_*c*_ and *v*_*0*_, and decreasing *λ*, the conventional CFRP-SHSCMs were prone to local bearing failure with poor ductility, leading to the insufficient use of the CFRP plate, in contrast, the improved CFRP-SHSCMs primarily underwent overall buckling failure and exhibited better bearing force and ductility. Finally, the modified Perry-Robertson formula was put forward to predict the ultimate load of the CFRP-SHSCMs. The coefficients of variation between the FE simulation and the theoretical results were 0.00436 and 0.0292, respectively.

## Introduction

Hollow steel sections are applied extensively in truss structures, steel frames, power transmission towers, and offshore platforms due to their beautiful appearance, high bearing force and ductility, and abundant section specifications^1–3^. And square hollow sections (SHS) are used as axially compressed members owing to their equal bending stiffness around two axes^4–6^. However, axially compressive SHS with large or medium slenderness ratios are prone to overall buckling failure, triggering a sharp decrease in bearing force and stiffness, which disrupts the control of the structural deformation. Therefore, many design codes from multiple countries specify the requirement for the diameter-thickness ratio (*D*/*t*) or the width-thickness ratio (*b*/*t*) of the square hollow sections, and this also leads to a dramatic increase in the weight and cost^7–9^. Moreover, commonly used steel structures also typically have difficulty meeting the requirements of engineering practice in complex and harsh environment, and usually require additional protection by functional materials. In summary, effectively improving the durability, ductility, and bearing force of slender square steel column without increasing weight is a critical issue that has received extensive attention in the recent research.

Fibre-reinforced polymers (FRPs) have been broadly used in building structures because of their excellent mechanical characteristics^10–19^. At present, many scholars have conducted related research on FRP reinforced steel column members. Gao X Y and Balendra^20^ explored the axial compressive behavior of circular steel column reinforced with CFRP sheets, focusing on the effect of wall thickness, slenderness ratio, and the number of CFRP layers. Their findings suggest that the use of CFRP can significantly enhance both the strength and stiffness of the brace. Through the investigation of the elephant's foot buckling strength in thin-walled steel columns reinforced with CFRP sheets^21,22^, it was discovered that FRPs can effectively enhance the local buckling strength of thin-walled steel columns. To promote the application of FRP strengthening technology, Ref.^23^ proposed transferring FRP material into steel by the equivalent stiffness method, and then, the transformed cross-section was calculated using the formula for conventional composite members. Kumar A P and Senthil R^24^ conducted a study on the mechanical performance of circular steel columns reinforced with CFRP under cyclic loading. Their findings indicated that CFRP can enhance the bearing force and ductility of the steel column. Nevertheless, local bearing failure at the end of the steel pipe was identified as a potential weakness, which compromised the overall bearing force and ductility of the composite member. To tackle this issue, Liu Z Q^25,26^ suggested incorporating thick-walled steel columns at both ends of the CFRP-circular steel tube composite member to improve the connection between the steel tube and CFRP sheet, and achieved satisfactory results in use. Considering the marked discrepancy between the mechanical performance of the circular and square steel columns, and between the CFRP sheet and plate, the findings of the previous study could not be directly applied to the CFRP plate reinforced square steel column composite members.

Shaat and Fam^27–30^ investigated the mechanical performance of CFRP plate reinforced steel square hollow sections under axial and eccentric compressive loads based on laboratory tests and parametric FE analysis. Their analysis indicated that the CFRP plate can significantly enhance the global buckling force of the axially compressive square steel hollow sections and avoid a drastic decrease in both the bearing force and the stiffness following the occurrence of specimen’s overall buckling. However, for a given slenderness ratio and initial out-of-straightness value of the composite member, it could occur that the bearing force of the specimens increase slightly as the amount of CFRP layers elevates, because the bearing force of the member is controlled by the local bearing failure at the end. Considering that the thick-walled section can efficiently ensure the bonding performance and the local bearing strength of the composite members^25,26^, thus, it is essential to explore the force mechanism of the improved CFRP plate-reinforced thin-walled square hollow section under the compressive load.

**Figure 1:**
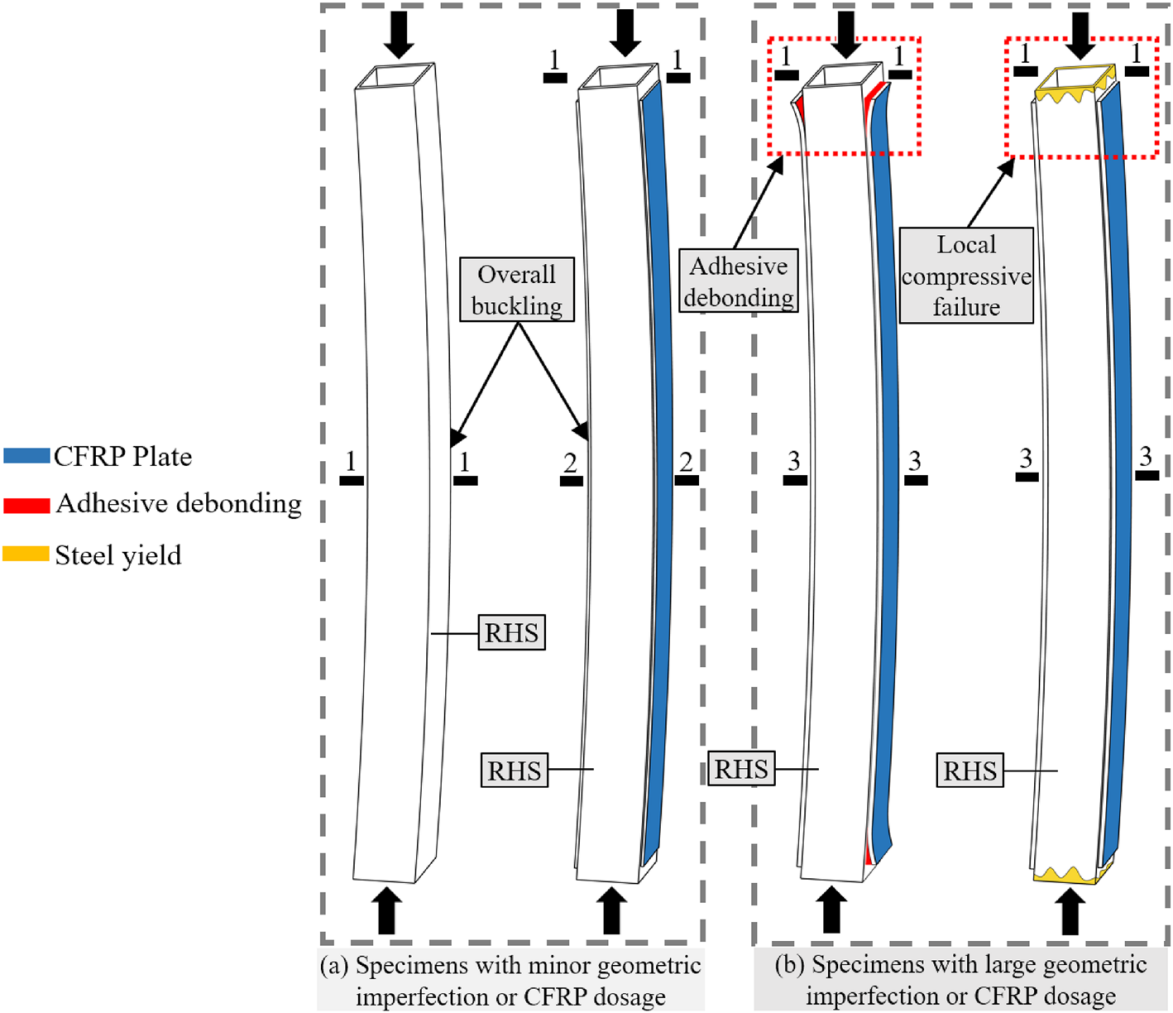
Typical failure modes of the CFRP plate-reinforced equal-thickness square hollow section. (Section 1-1: steel hollow section; Section 2-2: steel hollow section strengthened with thin CFRP layer; Section 3-3: steel hollow section strengthened with thick CFRP layer).

**Figure 2:**
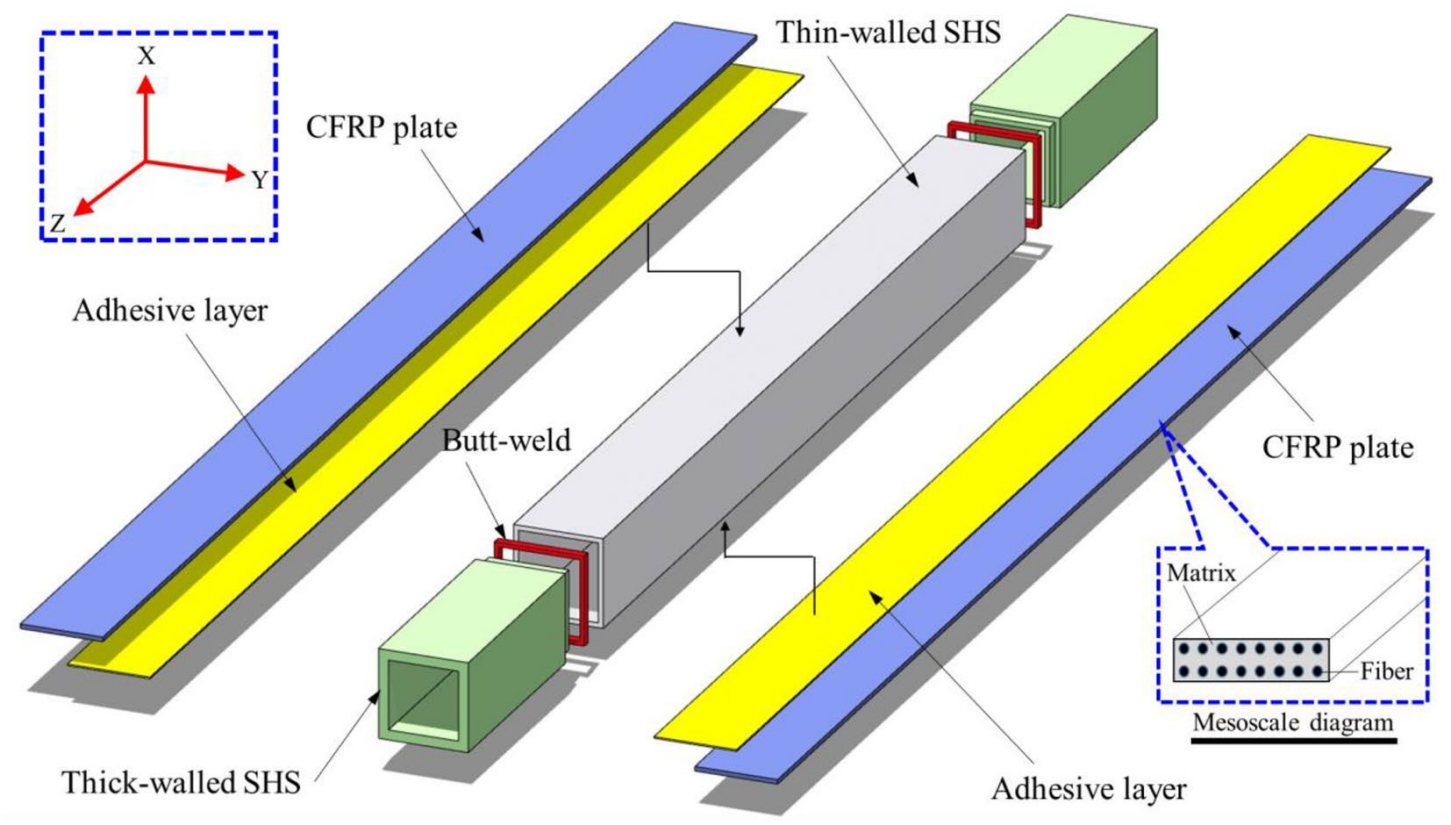
Sketch of the improved CFRP plate-strengthened square hollow section composite member.

The objective of this study is to introduce an improved CFRP plate-strengthened square hollow section composite member (CFRP-SHSCM) to prevent local bearing failure or debonding failure (Fig. 1b) by installing the thick-walled section at the end of the specimen. The sketch of the investigated CFRP-SHSCM is plotted in Fig. 2. The range and accuracy of the investigated parameters are limited by the manufacturing technique of the test specimens, and some related parameters, such as initial geometric imperfections (*v*_*0*_), cannot be accurately controlled or monitored experimentally. Therefore, the mechanical performance of the proposed CFRP plate-strengthened square hollow section composite member under axial compressive load is explored through a refined parametric FE analysis, and the amount of CFRP layers (*n*_*c*_), the initial geometric imperfection (*v*_*0*_), the slenderness ratio(*λ*), the CFRP layouts (2S and 4S) and the length of the exposed steel column (*L*_*e*_) were incorporated. The calculation formula of ultimate load of the CFRP-SHSCM was also proposed.

## FE modelling

### Details of the specimens

**Figure 3:**
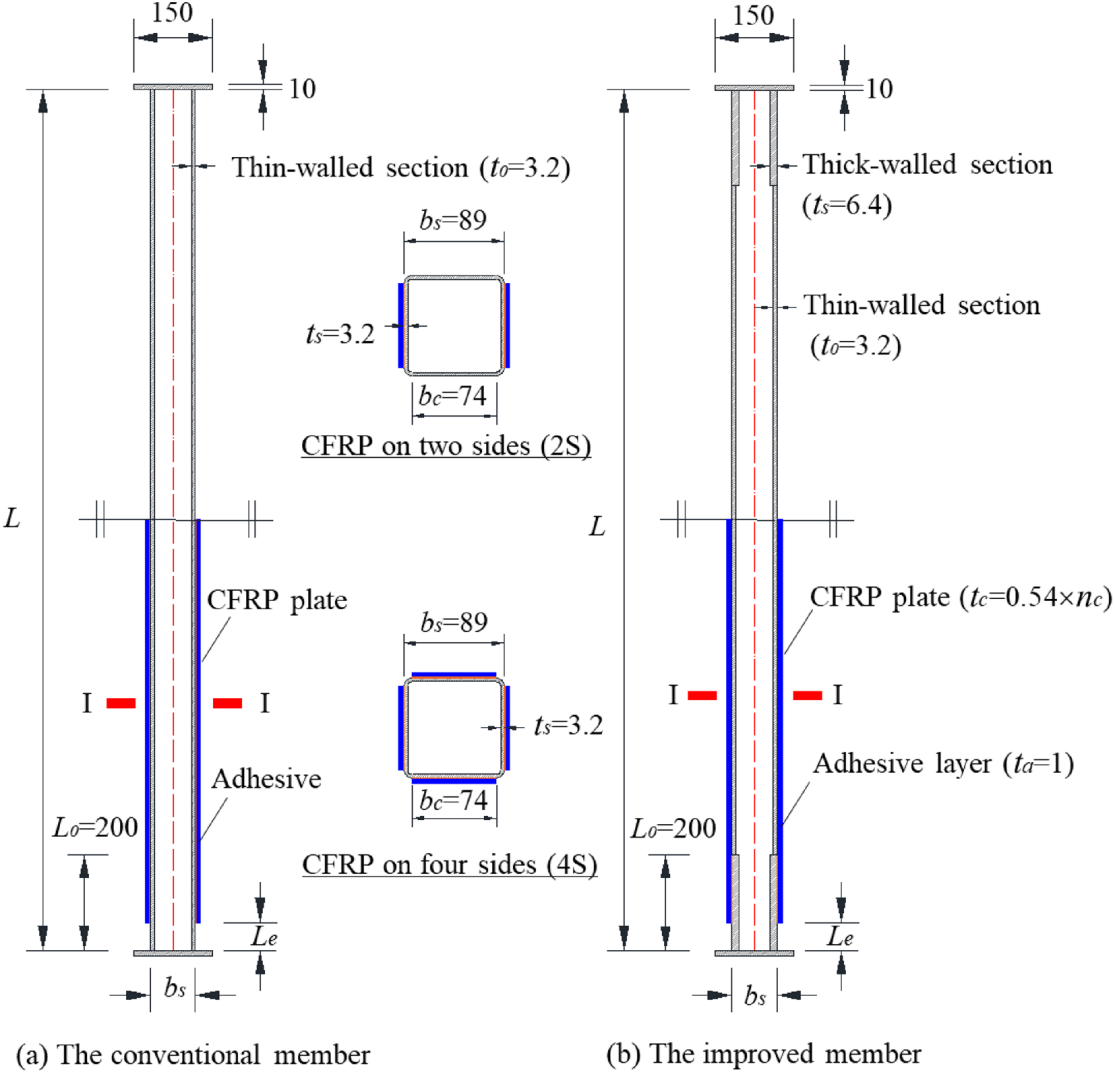
Configuration details of the CFRP-SHSCMs.

Figure 3 plotted the configuration details of the conventional and improved members. The conventional members all adopted the 89 × 89 × 3.2 mm thin-walled hollow steel section. The improved members consist of three parts, a 89 × 89 × 3.2 mm steel section column in the middle and two 89 × 89 × 6.4 mm thick-walled steel section columns with the length of 200 mm at both ends. The layout of CFRP plate is divided into two kinds of forms, bonded on two sides (2S) or four sides (4S) of steel column. Except for the members in Section "Effects of the length of the exposed steel column", the length of the exposed steel column at the end of the members in this paper is 25 mm.

### Material parameters

All square hollow sections (SHS) were cold-formed. According to Refs.^31,32^, the prior damage occurs at the thin-walled section of unequal-thickness steel columns connected by butt welds, and cast steel connectors also ensure that the welds do not fracture before the steel column under cyclic loading. Therefore, the material properties of the SHS were used for the butt-weld to simplify the FEM. The CFRP plate was a high-strength pultruded unidirectional fibre plate. The adhesive layer was composed of a two-component epoxy resin. The measured material properties of the related materials are taken from Ref.^27^, and are listed in Table 1.

**Table 1:** Material properties of the composite members.

Materials	Elastic modulus (*E*)			Passion ratio (*υ*)			Yield strength (*f*_*y*_)	Tensile strength (*f*_*t*_)	Elongation (*ε*_*t*_)	Shear strength (_*t*_)
	*Ex*	*Ey*	*Ez*	*υ*_*xy*_	*υ*_*yz*_	*υ*_*zx*_				
SHS	200 GPa			0.30			380MPa	–	0.25	–
CFRP	73.3 GPa	4.61 GPa	4.61 GPa	0.31	0.39	0.02	–	970MPa	0.01	14MPa
Adhesive	2.4 GPa			0.30			–	70MPa	0.05	24MPa

### Mesh generation

**Figure 4:**
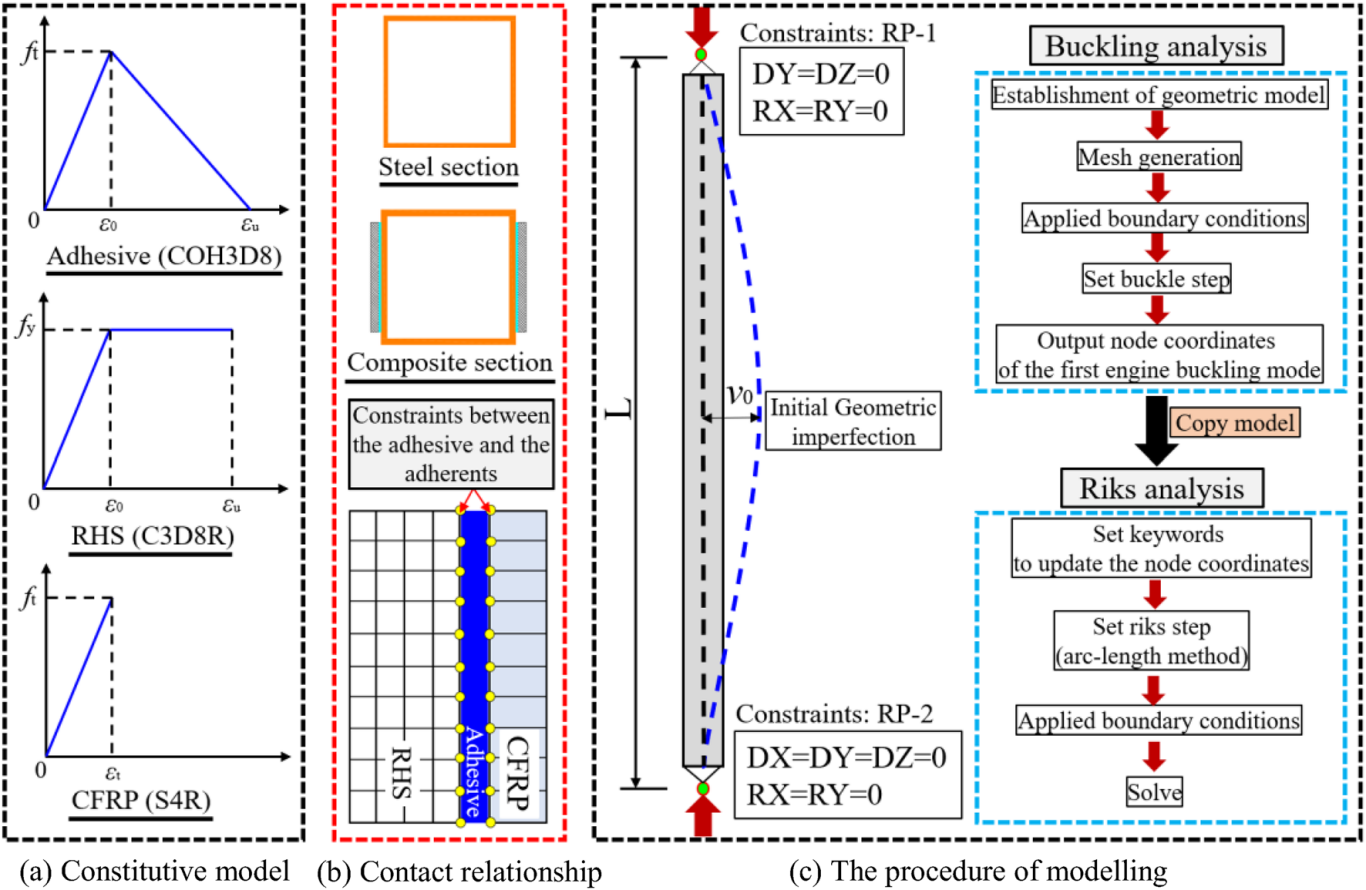
Schematic diagram of establishment of the FE model.

A FE simulation was conducted using the commercial finite element software ABAQUS_V14.0^33^. The rectangular hollow section (RHS) was modelled with the three-dimensional solid reduced integral element (C3D8R). The epoxy resin adhesive was simulated using the three-dimensional eight-node cohesive element (COH3D8). The CFRP plate was modelled by 4-node shell element (S4R), and its orthogonal anisotropy properties were incorporated into the FE model using the parameters provided by the manufacturer. Figure 4a and Fig. 4b exhibit the constitutive model of the related materials and the contact relationship among each part, respectively. The specific procedure of modelling is shown in Fig. 4c. Two reference points (RPs) were set at both ends of the CFRP-SHSCM, and coupled with the sidewall of the steel tube at the respective position. The boundary conditions and the axial compression loading are applied to the RPs. Eigenvalue buckling analysis was performed on the FE model to derive the first-order buckling mode. Then, the engine buckling deformation shape was introduced into the FE model, and the node coordinates of the CFRP-SHSCM were updated. Finally, nonlinear buckling analysis (Riks analysis) was performed on the FE model containing the initial geometric imperfections.

### Verification of FE modelling

**Figure 5:**
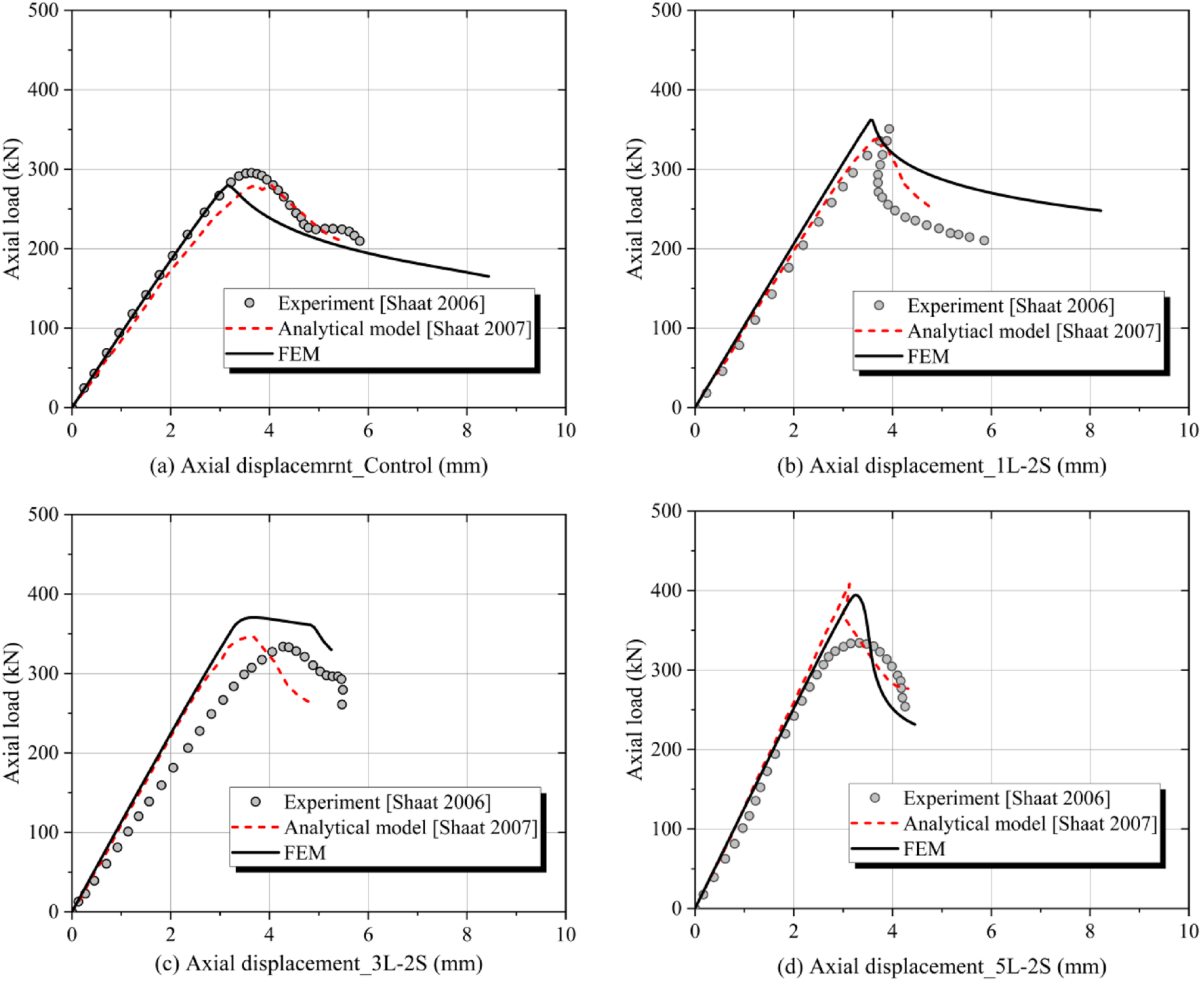
Validation of the FEM.

To verify the accuracy of the adopted finite element model (FEM), both the numerical and experimental results of the test members in the literature^27,28^ were compared with the simulation results of this FEM. Figure 5 shows the comparative results of load-vertical displacement curves of the test members, including a control specimen and three composite specimens strengthened with one, three, and five layers of CFRP plates applied on two opposite sides, respectively. The load–displacement curve derived from the used FEM is closer to that obtained from the analytical model in the literature^28^. In the finite element model adopted, the mesh size of the RHS is 10 mm, the FRP is 20 mm, and the adhesive layer is 10 mm. Only one layer of mesh is set along the thickness direction of the adhesive layer and the FRP. Four layers of mesh are set along the thickness direction of the RHS. And a difference (within 5%) is observed between the FEM and experimental data, which is primarily due to the initial eccentricity of the applied load, the uncertainty of the initial geometric imperfection of the test members, and the accidental error of the test device. Considering that the error is acceptable, therefore, the used FEM can accurately simulate the mechanical characteristics of the CFRP-SHSCM.

## Mechanical performance of the CFRP-SHSCMs

### Geometric parameters

According to Ref.^28^, the investigated factors such as the amount of CFRP layers (*n*_*c*_), the initial geometric imperfection (*v*_*0*_), and the slenderness ratio of the member (*λ*) have a substantial impact on the bearing force, typical failure modes, and ductility of the CFRP-SHSCMs. Therefore, a comparative analysis is first conducted among the load–displacement relationship and the Mises stress distribution of the eight conventional and improved CFRP-SHSCMs (*L* = 2380 mm; *v*_*0*_ = 1/1000*L*).

#### Load‒displacement relationship

**Figure 6:**
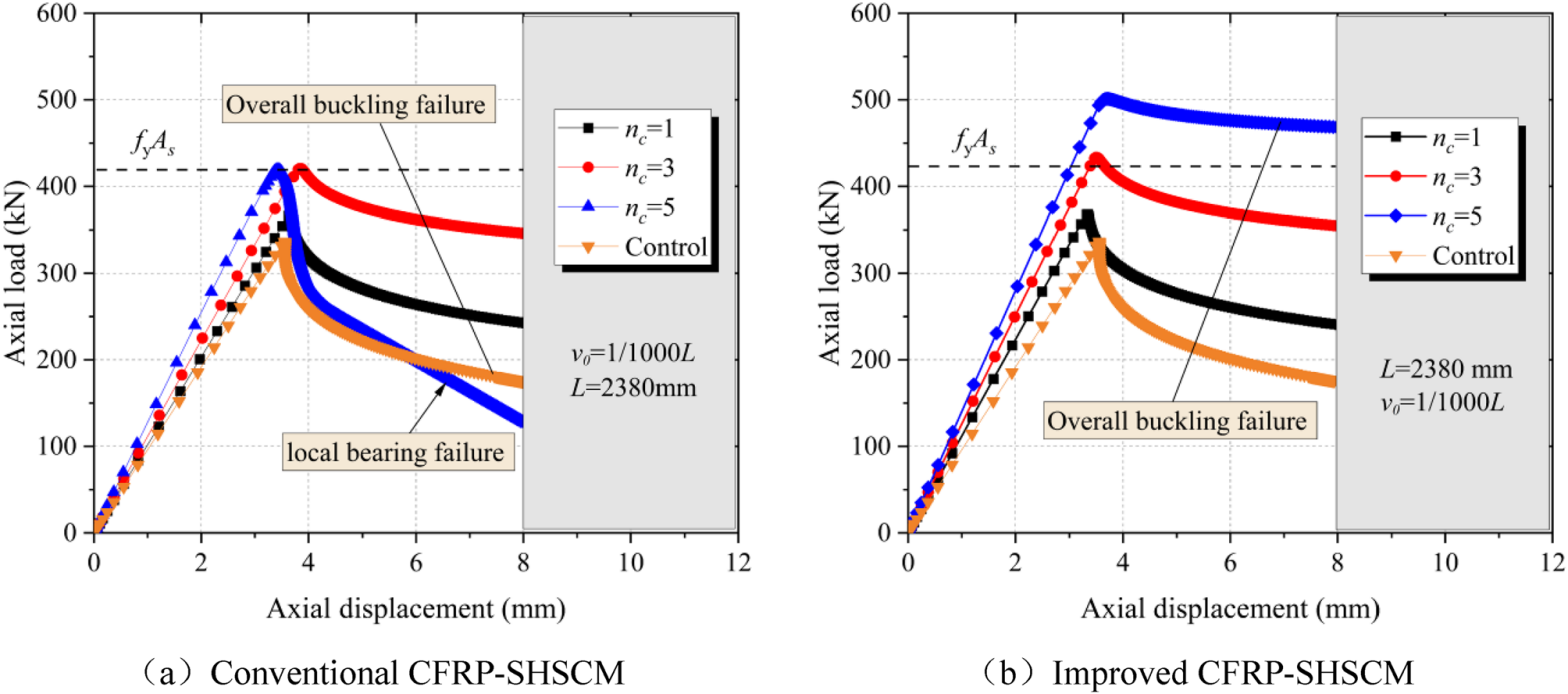
Axial load–displacement curves of the CFRP-SHSCMs.

Figure 6 shows the load-vertical displacement curves for the conventional and the improved CFRP-SHSCMs across varying amount of CFRP layers (*n*_*c*_ = 0, 1, 3 and 5), and the corresponding ultimate load of the members are summarized in Table 2. For the conventional CFRP-SHSCMs, when *n*_*c*_ increases from 0 to 3, the members all undergo overall buckling failure, and the ultimate load also increases compared to *n*_*c*_ = 0, with a maximum increase of 25.01%. When *n*_*c*_ elevates from 3 to 5 layers, the conventional CFRP-SHSCM occurs the local bearing failure, and the ultimate load of even decreases slightly by 1.3kN and the bearing force and stiffness of the member decrease more sharply after the local bearing failure occurs. Thus, for the CFRP strengthened equal thickness composite members, when *n*_*c*_ increases to a certain amount, the bearing force of the members is constrained by the local bearing failure occurs at the end of the steel column. From Fig. 6b, the improved CFRP-SHSCMs all experience overall buckling failure (*n*_*c*_ = 0 ~ 5,), and the ultimate load of the improved CFRP-SHSCM gradually increases as *n*_*c*_ rises with a maximum increase ratio of 48.93%. It shows that the improved CFRP-SHSCMs effectively avoids local bearing failure by setting thick-walled steel section at the end, ensuring the continuous growth of the ultimate load. Moreover, by comparing the ultimate load of the conventional and improved CFRP-SHSCM, it can be found that when *n*_*c*_ = 1 and 3, the two specimens are almost equal, and the improved CFRP-SHSCM is approximately 21.1% higher than that of the conventional CFRP-SHSCM at *n*_*c*_ = 5, and its bearing force and stiffness decrease more slowly after reaching the ultimate load. As a result, the improved CFRP-SHSCM exhibits more superior mechanical behavior compared to the conventional CFRP-SHSCM.

**Table 2:** The ultimate load of the CFRP-SHSCM while *L* = 2380 mm; *v*_*0*_ = 1/1000*L*.

Type	Label	*n*_*c*_	Failure mode	*P* (kN)	*β*_*1*_ = P*/*C-1(I-1)	*β*_*2*_ = I-i */*C-i
Conventional CFRP-SHSCM	C-1	0	OB	336.5	–	–
	C-2	1	OB	367.4	9.20%	–
	C-3	3	OB	420.6	25.01%	–
	C-4	5	LB	419.3	24.62%	–
Improved CFRP-SHSCM	I-1	0	OB	337.1	–	0.18%
	I-2	1	OB	368.0	9.37%	0.15%
	I-3	3	OB	433.4	28.81%	3.04%
	I-4	5	OB	501.1	48.93%	19.51%

OB denotes the overall buckling failure; LB denotes the local bearing failure; i=1 ~ 4.

#### Failure modes of the CFRP-SHSCMs

**Figure 7:**
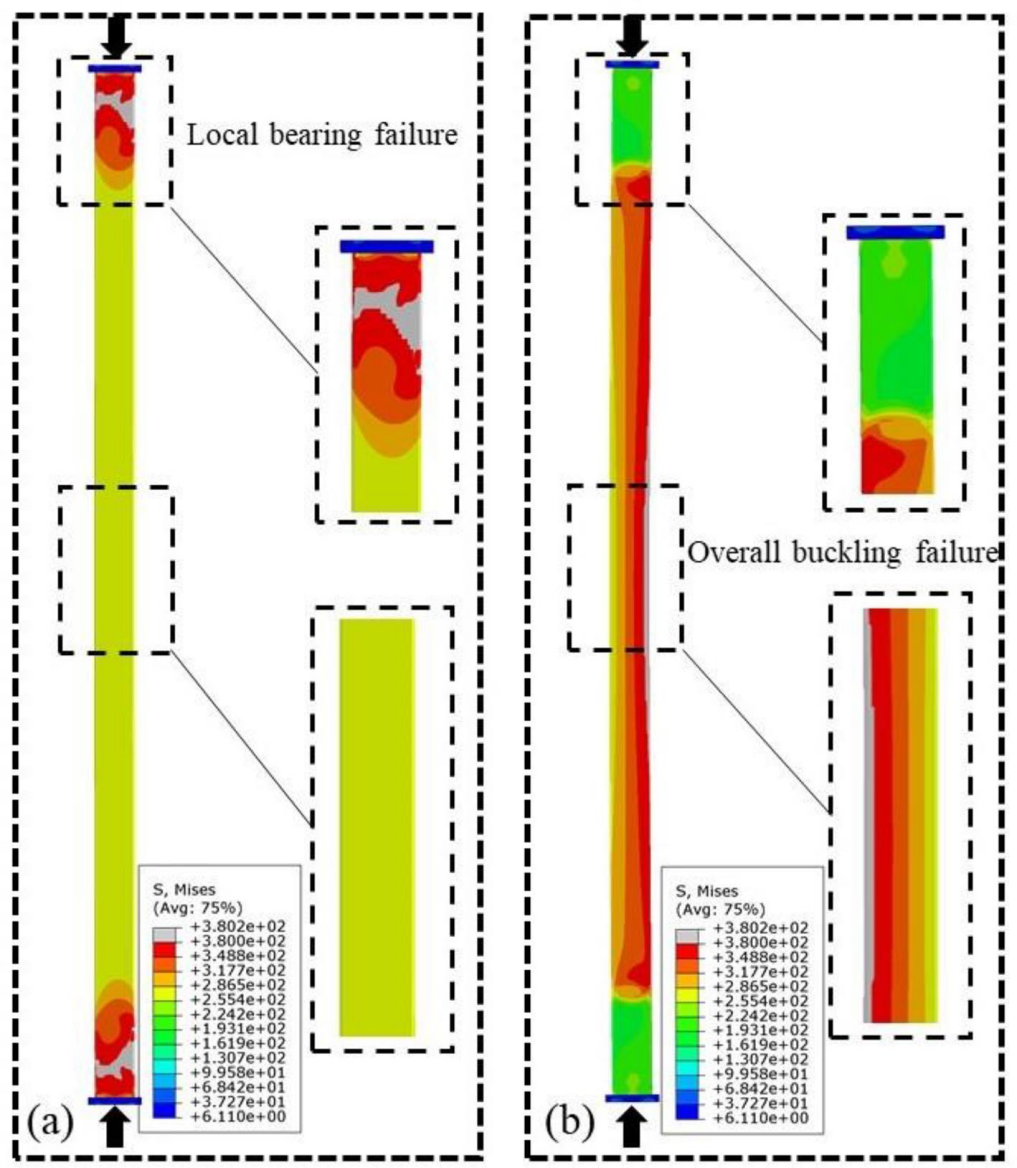
Mises stress nephograms of the steel column at the peak load (*n*_*c*_ = 5; *L* = 2380 mm; *v*_*0*_ = 1/1000*L*).

When the ultimate load is achieved, the Mises stress distributions of steel column of CFRP-SHSCMs (*n*_*c*_ = 5; *L* = 2380 mm; *v*_*0*_ = 1/1000*L*) are displayed in Fig. 7. The conventional CFRP-SHSCMs experience local bearing failure at the end of the steel column (Fig. 7a), resulting in the strength of the built-up section not being sufficiently exerted. The improved CFRP-SHSCMs experience overall buckling failure at the middle of the member (Fig. 7b), which is mainly due to the fact that the overall buckling bearing force of the built-up section surpasses the local bearing force of the steel column at the end. Therefore, the thick-walled steel column can considerably enhance the local carrying capacity of the end of the CFRP-SHSCM to avoid the occurrence of the local bearing failure of the steel column, and the improved CFRP-SHSCMs exhibit a higher bearing force and reasonable failure modes.

## Parametric analysis

### Effects of the initial geometric imperfection

Design specifications from multiple countries require initial geometric imperfections (*v*_*0*_) to be less than 1/1000*L* for structural steel members. Considering that the initial geometric imperfections are random values that cannot be precisely controlled in engineering practice, it is necessary to study the effect of the initial geometric imperfections on the mechanical performance of the CFRP-SHSCMs. Therefore, a total of 72 conventional and improved CFRP-SHSCMs were designed. According to the difference in the amount of CFRP layers (i.e., *n*_*c*_ = 3 and 5) and the slenderness ratio (i.e., *L* = 2380, 2808, and 3276 mm), the conventional and improved CFRP-SHSCMs were divided into six groups, respectively. The variables in each group were the initial geometric imperfection (i.e., *v*_*0*_ = 1/16000*L*, 1/8000*L*, 1/4000*L*, 1/2000*L*, 1/1000*L* and 1/500*L*).

**Figure 8:**
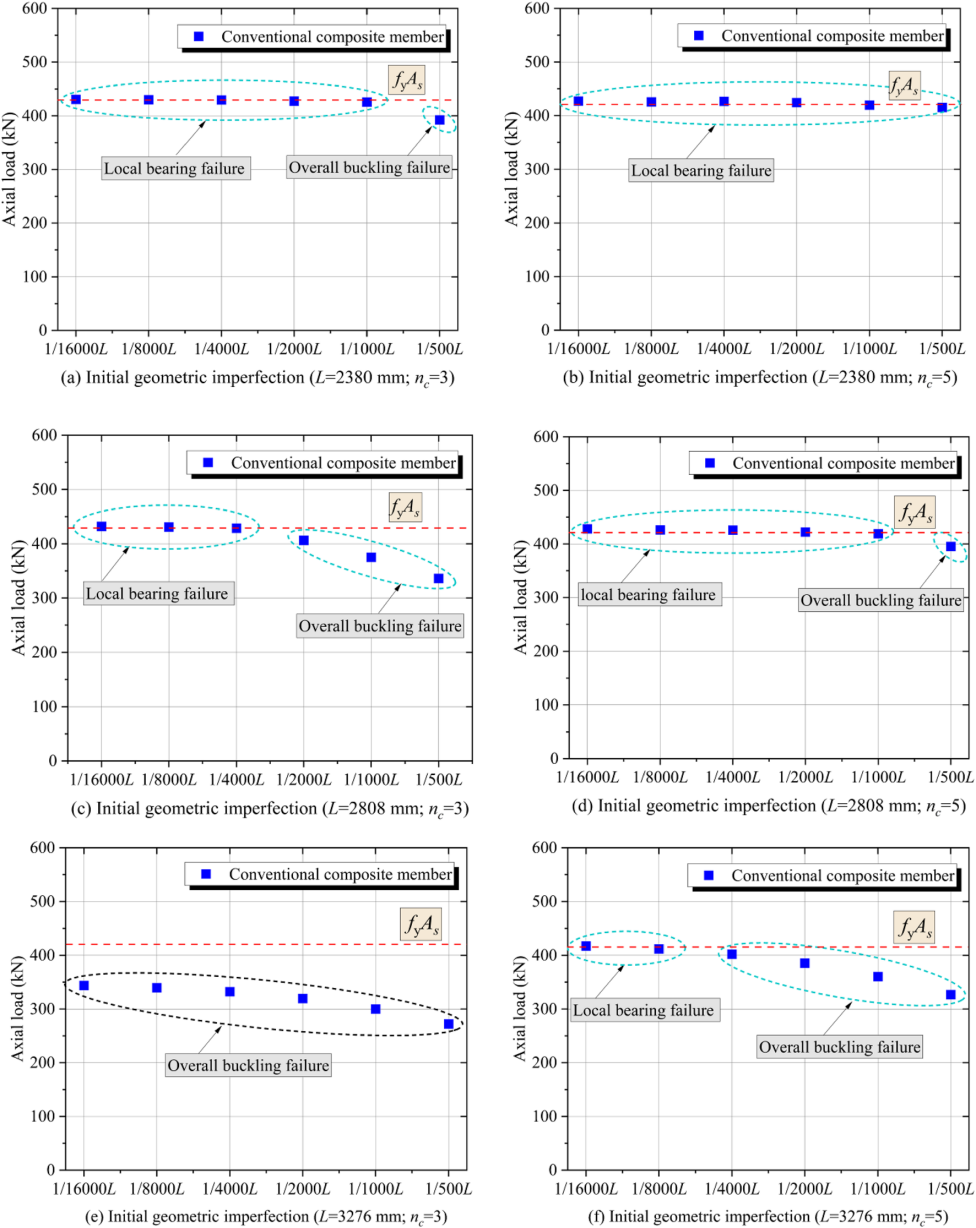
Bearing force and typical failure modes of the conventional CFRP-SHSCMs.

The bearing force and typical failure modes of 36 conventional composite components are plotted in Fig. 8. When *L* = 2380 mm and *n*_*c*_ = 3 (Fig. 8a), the overall buckling failure occurs at the middle (*v*_*0*_ = 1/500*L*), and the local bearing failure occurs at the end of the member (*v*_*0*_ ≤ 1/1000*L*). Thus, it can be concluded that for the conventional CFRP-SHSCM, *v*_*0*_ has a great effect on the overall buckling bearing force, but has a limited effect on the local bearing force at the loading end. And the relative relationship between the overall buckling and the local bearing force at the end determines the failure mode of the member. When *n*_*c*_ increases from 3 to 5, the failure mode of the CFRP-SHSCMs changes from the overall buckling failure to the local bearing failure at *v*_*0*_ = 1/500*L*, indicating that although the overall buckling bearing force of the middle section of the specimen is increased, the local bearing force of the steel column at the end limits the full use of the built-up section. When *L* = 3276 mm and *n*_*c*_ = 3 (Fig. 8e), the CFRP-SHSCMs all experience overall buckling failure. The results reveal that the conventional CFRP-SHSCMs are more likely to undergo overall buckling failure as the slenderness ratio elevates. Based on the aforementioned analysis, the typical failure modes of conventional CFRP-SHSCMs are influenced by the combination of the amount of CFRP layers, slenderness ratio and initial geometric imperfection.

**Figure 9:**
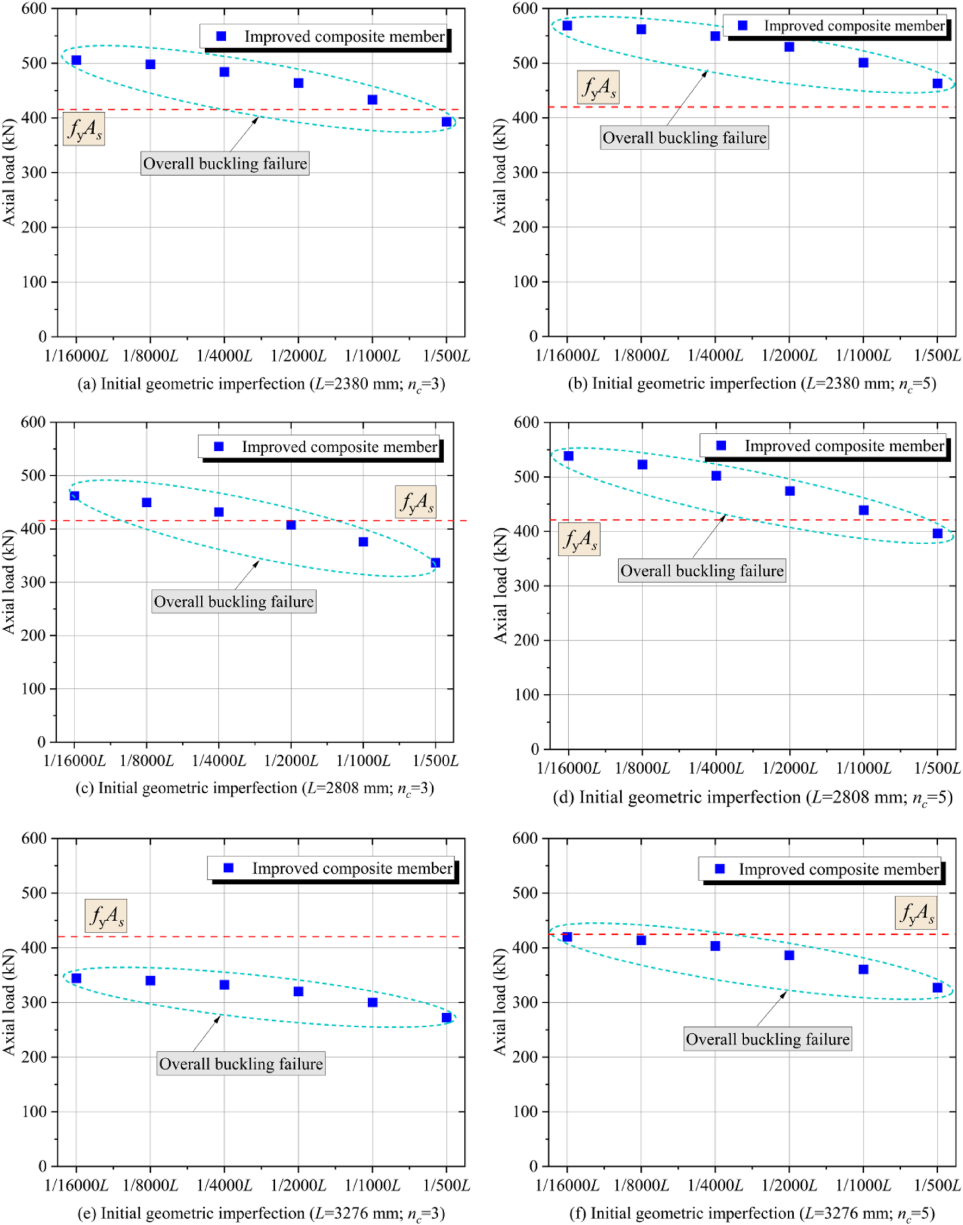
Bearing force and typical failure modes of the improved CFRP-SHSCMs.

Figure 9 depicts the typical failure modes and the corresponding bearing force of the improved CFRP-SHSCMs with the variation of the amount of CFRP layers, the slenderness ratio, and the initial geometric imperfections. The failure modes of the improved CFRP-SHSCMs are all overall buckling failure. When the improved and the corresponding conventional CFRP-SHSCMs both suffer from the overall buckling failure, they have the same bearing force. While the improved CFRP-SHSCMs exhibit a higher bearing force compared to the corresponding conventional CFRP-SHSCMs that experience local bearing failure. Thus, the bearing force of the improved CFRP-SHSCM is not limited by the local bearing force of the steel column. Moreover, by comparing the bearing force of the specimens (Fig. 9a ~ 9f.), it can be observed that the bearing force of the improved CFRP-SHSCM gradually decreases with an increase in *v*_*0*_ and *L*, and gradually increases with an increase in *n*_*c*_. Table 3 depicts that among the six groups of members, when* L* = 2808 mm and *n*_*c*_ = 3, the maximum reduction in bearing force of the member is 28.10% with *v*_*0*_ = 1/500*L* compared to *v*_*0*_ = 1/16000*L*.

**Table 3:** The maximum reduction of axial load.

No	*L*(mm)	*n*_*c*_	P_1_ (kN)	P_2_ (kN)	△
1	2380	3	500	395	21.00%
2	2808	3	452	325	28.10%
3	3276	3	340	275	19.12%
4	2380	5	560	450	19.64%
5	2808	5	530	394	25.66%
6	3276	5	410	320	21.95%

P_1_—Maximum axial load; P_2_—Minimum axial load; △ = ((P_1_−P_2_)/P_1_) %.

### Effects of the layout of the CFRP plate

**Figure 10:**
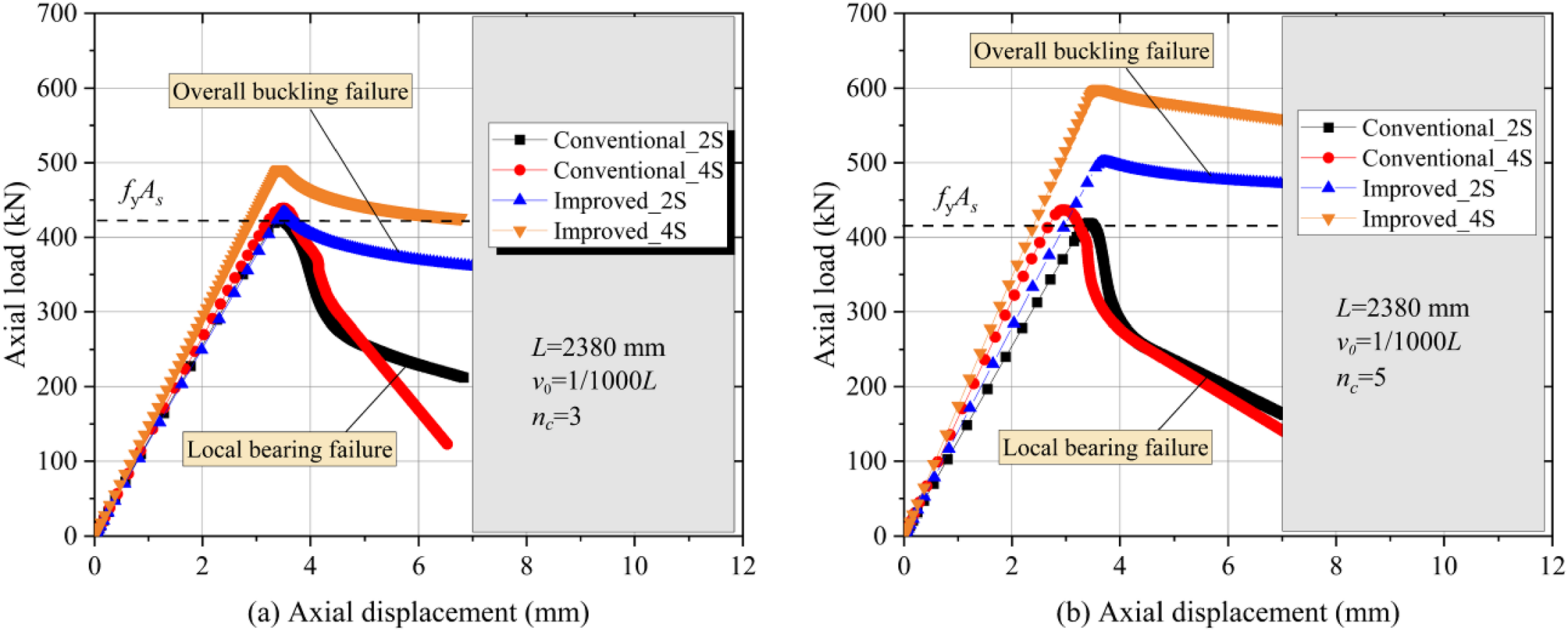
Effects of CFRP layout on the load‒displacement relationship.

According to Refs.^27,29^, the overall buckling bearing force of members with CFRP applied on all four sides (4S) of a square steel hollow section is higher than that of the specimens equipped with CFRP on two opposite sides (2S). Therefore, it is necessary to investigate the conventional and improved CFRP-SHSCMs equipped with CFRP on all four sides. Figure 10 shows the load–displacement curves of the two types of CFRP-SHSCMs when *n*_*c*_ is 3 (Fig. 10a) and 5 (Fig. 10b), and their ultimate loads are summarized in Table 4. For conventional CFRP-SHSCMs, the use of CFRP bonded on all four sides cannot effectively improve the bearing force of the members, which is primarily due to the local bearing failure of the steel column, limiting the fully utilization of the built-up section. However, for the improved CFRP-SHSCMs, when CFRP is bonded on all four sides, the bearing force and initial stiffness markedly improve compared to the CFRP bonded on both sides. And when *n*_*c*_ = 3 and 5, the ultimate load of the improved CFRP-SHSCM equipped with CFRP on four sides are 13.34% and 19.59% higher than that of the corresponding member equipped with CFRP on two opposite sides, respectively. Based on the aforementioned analysis, the effect of the bearing force and initial stiffness of the conventional and improved CFRP-SHSCMs are not identical when CFRP is applied on all four sides.

**Table 4:** The ultimate load of the CFRP-SHSCM while *L* = 2380 mm; *v*_*0*_ = 1/1000*L.*

Type	*n*_*c*_	CFRP layout	Failure mode	*P* (kN)	*β* = 4S*/*2S
Conventional	3	2S	LB	426.6	–
	3	4S	LB	438.9	2.89%
Improved	3	2S	OB	433.4	–
	3	4S	OB	491.2	13.34%
Conventional	5	2S	LB	419.3	–
	5	4S	LB	436.8	4.17%
Improved	5	2S	OB	501.0	–
	5	4S	OB	599.1	19.59%

### Effects of the length of the exposed steel column

According to the previous analysis, the bearing force of the conventional CFRP-SHSCM is determined by the local bearing force of the steel column at the end. Therefore, it is important to study the effect of the length of the exposed steel column (*L*_*e*_) on the ultimate load of the conventional CFRP-SHSCM, with both geometric imperfection and the layout of the CFRP plate taken into account.

**Figure 11:**
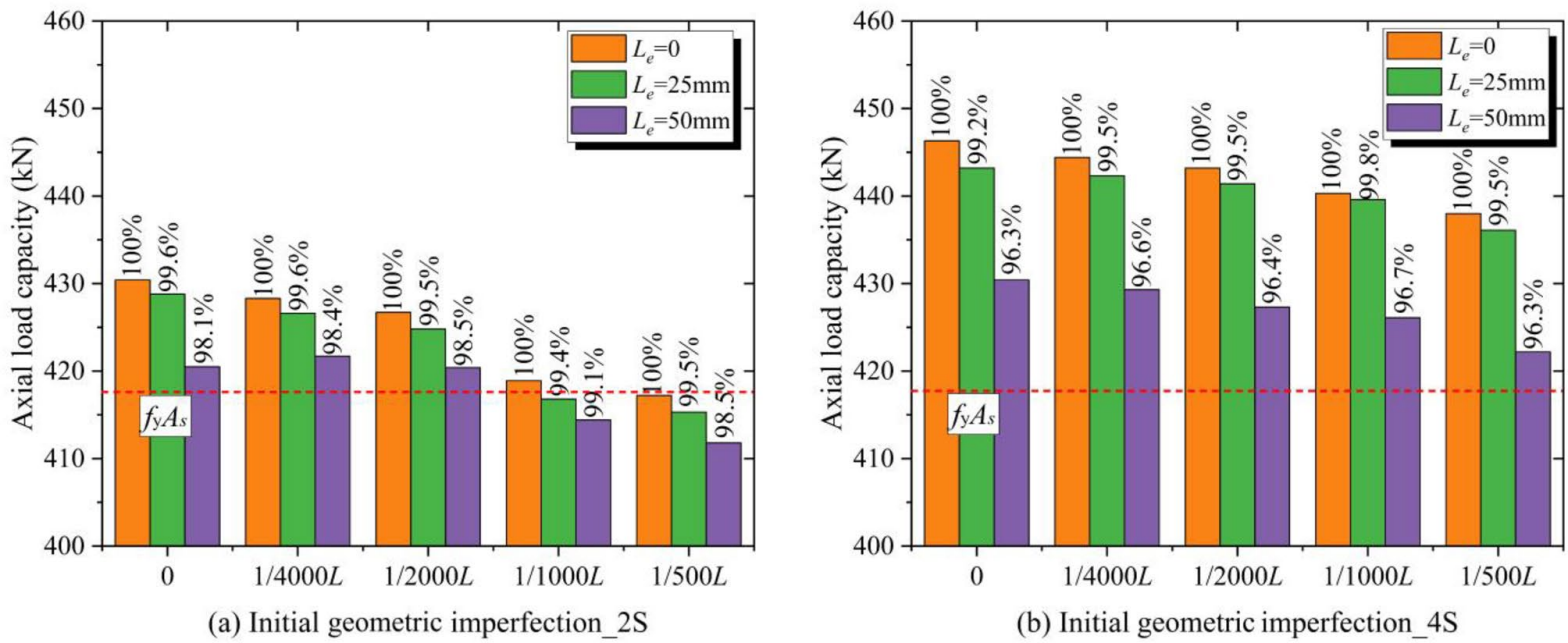
The bearing force of the conventional CFRP-SHSCM (*n*_*c*_ = 5; *L* = 2380 mm).

The effect of initial geometric imperfection (*v*_*0*_), the length of the exposed steel column (*L*_*e*_ = 0 ~ 50 mm), and the layout of CFRP plate (4S and 2S) on the ultimate load of the conventional CFRP-SHSCMs (*L* = 2380 mm; *n*_*c*_ = 5) is shown in Fig. 11. The members all undergo local bearing failure at the end of the steel column. Under the different *v*_*0*_, the local bearing force of the member all decreases gradually slightly with the increase in *L*_*e*_. When *L*_*e*_ = 50 mm, the maximum decrease of the bearing force of the member with CFRP bonded on both sides is 1.9%, and that of the member with CFRP applied on four sides is 3.7%. Thus, *L*_*e*_ has a limited effect on the local bearing force of the conventional CFRP-SHSCM, which demonstrates the local bearing force is mainly provided by the steel column at the end rather than the CFRP plate. Even if CFRP plates are installed on all four sides of the steel column, it cannot change the fact that the end of steel column is the weak part of the specimen. Therefore, installing the thick-walled section at the ends can effectively enhance the connection strength of the steel column, resulting in a more reasonable failure mode and greater bearing force of the specimen.

## Axial compressive loaded capacity of the CFRP-SHSCMs

### Basic assumptions

The adhesive layer can be simplified into one unique layer in the theoretical analysis due to its limited effect on the bearing force of the member, as shown in^26^. Considering that the thickness of the CFRP plate is greater than that of the CFRP sheet, the plastic development of the steel column must be considered in the theoretical analysis. Therefore, the following assumptions are used to simplify the computational model:

1. Substitute multiple layers of CFRP plates into one layer of CFRP according to thickness;
2. The thickness of the adhesive layer is transformed by *t*_*a*_ = 0.5 (1 + *n*_*c*_) mm;
3. Substitute CFRP as elastic steel based on the flexural stiffness equivalent;
4. The distribution of the longitudinal strain in the equivalent section follows the plane section assumption;
5. The plastic development of the compressive wall of the steel hollow section is incorporated.

**Figure 12:**
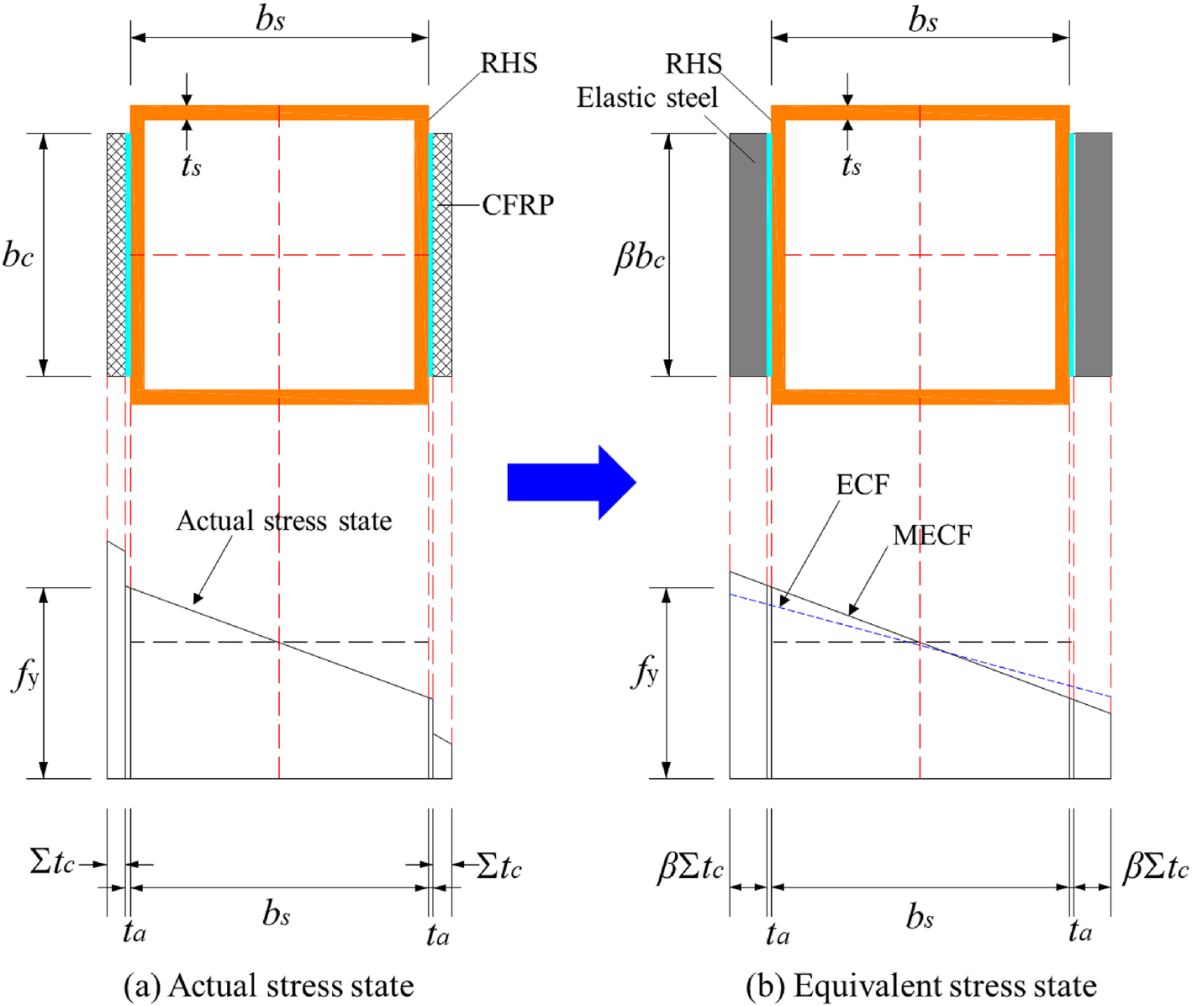
Diagram of the stress state of the equivalent section.

A modified formula for measuring the overall buckling bearing force of the built-up section was obtained according to the Perry-Robertson formula. Figure 12 shows the real state of the stress distribution and equivalent stress distribution of the built-up section (taking the example of bonding CFRP on two opposite sides of the square hollow section).

### Prediction of the overall buckling bearing force

According to the stress characteristics of the built-up section, the equivalent area (*A*_*t*_) and the equivalent moment of inertia (*I*_*t*_) can be obtained:

1 $$A_{t} { = }A_{s} + \beta A_{c} = A_{s} + \frac{{E_{c} }}{{E_{{\text{s}}} }}b_{c} t_{c}$$

According to Ref.^26^, the bending stiffness of the built-up section (bonded on both sides and all sides) can be expressed respectively as:

2 $$\left( {EI} \right)_{t} = \left\{ {\begin{array}{*{20}l} {\frac{{E_{s} }}{12}\left\{ {b_{{\text{c}}} \left[ {\left( {b_{s} + 2\beta n_{c} t_{c} } \right)^{3} - \left( {b_{s} + 2t_{a} } \right)^{3} } \right] + \left[ {\left( {b_{s} } \right)^{4} - \left( {b_{s} - 2t_{s} } \right)^{4} } \right]} \right\}} \hfill \\ {\frac{{E_{s} }}{12}\left\{ {b_{{\text{c}}} \left[ {\left( {b_{s} + 2\beta n_{c} t_{c} } \right)^{3} - \left( {b_{s} + 2t_{a} } \right)^{3} } \right] + \left[ {\left( {b_{s} } \right)^{4} - \left( {b_{s} - 2t_{s} } \right)^{4} } \right] + \left[ {\left( {2\beta n_{c} t_{c} } \right)\left( {b_{c} } \right)^{3} } \right]} \right\}} \hfill \\ \end{array} } \right.$$

where *A*_*c*_ and *I*_*c*_ represent the equivalent area and equivalent moment of inertia of the CFRP layers of the built-up section, respectively.

According to Ref.^26^, the global buckling coefficient *φ*_*t*_ of the improved CFRP-square steel column CFRP-SHSCM can be obtained:

3 $$\varphi_{{\text{t}}} = \frac{1}{2}\frac{{\left( {1 + \frac{1}{2}\left( {b_{s} - 2t_{s} } \right)\nu_{0} } \right)}}{{\overline{\lambda }_{{\text{t}}}^{2} }}{ - }\sqrt {\frac{1}{4}\left[ {1 + \frac{{\left( {1 + \frac{1}{2}\left( {b_{s} - 2t_{s} } \right)\nu_{0} } \right)}}{{\overline{\lambda }_{{\text{t}}}^{2} }}} \right]^{2} { - }\frac{1}{{\overline{\lambda }_{{\text{t}}}^{2} }}}$$

where:

4 $$\overline{\lambda }_{{\text{t}}} = \sqrt {\frac{{f_{{\text{y}}} }}{{\sigma_{{\text{e}}} }}} = \lambda_{t} \sqrt {\frac{{f_{{\text{y}}} }}{{\pi^{2} E_{s} }}}$$

5 $$\lambda_{{\text{t}}} { = }\frac{{L{\prime} }}{{\sqrt {{{I_{t} } \mathord{\left/ {\vphantom {{I_{t} } {A_{t} }}} \right. \kern-0pt} {A_{t} }}} }}{ = }\frac{\mu L}{{\sqrt {{{I_{t} } \mathord{\left/ {\vphantom {{I_{t} } {A_{t} }}} \right. \kern-0pt} {A_{t} }}} }}$$

Therefore, the global buckling capacity of the axially compressive CFRP-SHSCM can be expressed as:

6 $$P_{cr} \le f_{y} \varphi_{t} A_{t}$$

*P*_*cr*_ should also meet the limits of Eq. (7). Otherwise, *A*_*0*_ should be increased until the local carrying capacity of the CFRP-SHSCM is higher than the overall buckling bearing force:

7 $$P_{cr} \le f_{y} A_{0}$$

where *P*_*cr*_ is the ultimate load of the axially compressive CFRP-SHSCM, and *f*_*y*_ is the yield strength of the steel column.

### Verification of calculated results

**Figure 13:**
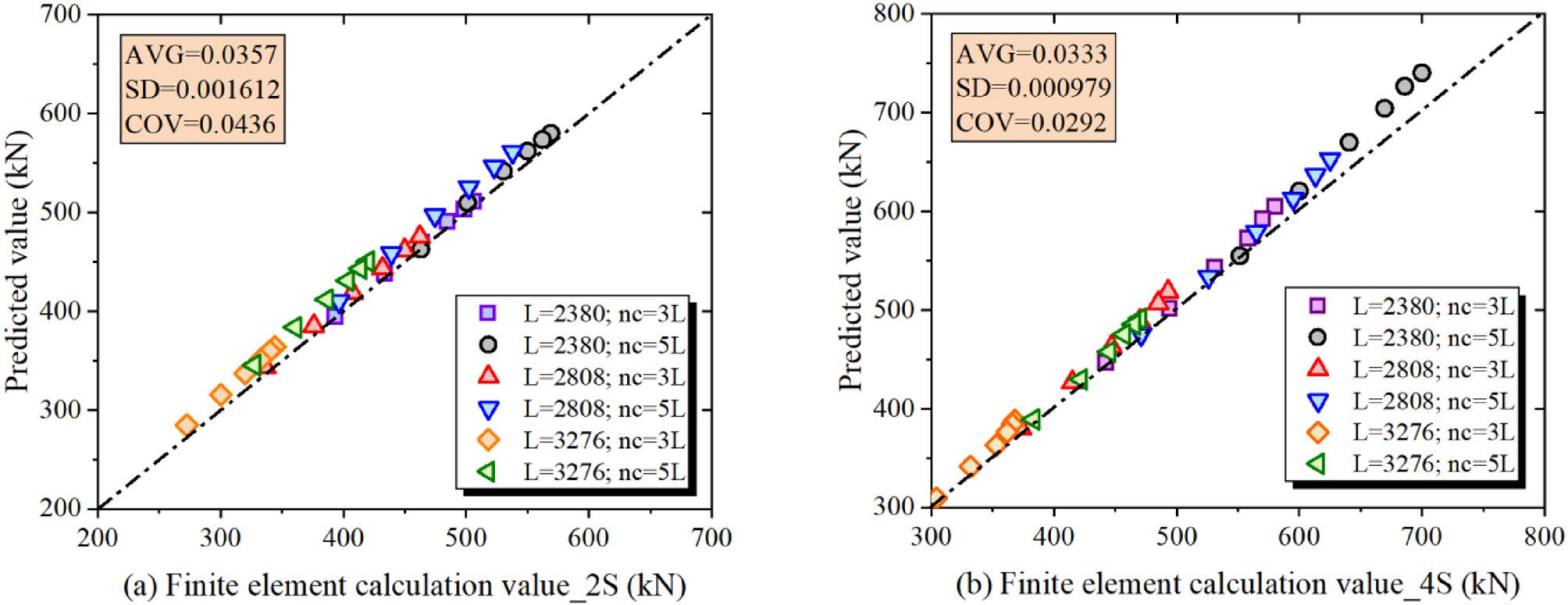
Comparison of the theoretical and FEM values of the improved CFRP-SHSCMs.

Parameters such as the initial geometric imperfection (*v*_*0*_) and the thickness of the adhesive layer (*t*_*a*_) of the test specimens are difficult to measure accurately. The error of the loading system is also difficult to calculate precisely for full-size specimens. The refined FEM can effectively simulate the mechanical characteristic of the composite specimens under axially compressive loading, including parameters such as the amount of CFRP layers, the initial geometric imperfections, and the slenderness ratio of the member. Therefore, the results of the finite element model (the improved CFRP-SHSCMs with CFRP bonded on both sides and four sides) were used to validate the theoretical results, and the verification data are presented in Fig. 13. According to the comparative findings, the maximum deviation of the FEM calculated results from the theoretical formula is only 7.23%, which occurs in the specimen with *L* = 3276 mm and *n*_*c*_ = 5. The coefficient of variation (COV) of the error of the calculation result is 0.0436 and 0.0292, respectively. The proposed formula can precisely estimate the axially compressive overall buckling bearing force of the CFRP-SHSCM.

## Conclusions

The study investigates the mechanical performance of both conventional and improved CFRP-SHSCMs under axially compressive loads using refined FE models. It comprehensively examines typical failure modes, Mises stress distribution, bearing force, and load-displacement relationships of the CFRP-SHSCMs. Additionally, and the influence of slenderness ratio and other factors on the mechanical characteristics of CFRP-SHSCM is analyzed. The key conclusions drawn from this study are as follows:

1. Conventional CFRP-SHSCMs are susceptible to local bearing failure, leading to an underutilization of the composite part's strength. Conversely, the improved CFRP-SHSCM, equipped with a thick-walled section at both ends, effectively harnesses the strength of the built-up section, enhancing bearing force and member ductility.
2. The overall buckling bearing force of the CFRP-SHSCM diminishes with increasing initial geometric imperfections and slenderness ratio, while it rises with additional layers of CFRP. However, these parameters exhibit limited influence on the local bearing force at the member's end.
3. The layout of CFRP plate and the length of the exposed steel column has a limited effect on the local bearing force of the conventional CFRP-SHSCM.
4. The Perry-Robertson formula was modified to estimate the ultimate load of the CFRP-SHSCMs. The FEM calculated results of the improved CFRP-SHSCMs were compared with the theoretical formula with a maximum deviation of 7.23%.

## Data Availability

All data generated or analysed during this study are included in this manuscript.

## Abbreviations

*E**CS*: Equivalent built-up section
*MECS*: Modified equivalent built-up section
SHS: Square hollow section
2S/4S: CFRP bonded on two opposite sides or four sides
*I*_*t*_: Inertia moment of the combined cross-section
*b*_*s*_: Width of the square hollow section
*b*_*c*_: Width of the CFRP plate
*t*_*s*_: Wall-thickness of square hollow section
*E*_*s*_: Steel elastic modulus
*E*_*c*_: Elastic modulus of CFRP plates along the fibre direction
*n*_*c*_: Number of CFRP layer
*t*_*c*_: CFRP plates thickness
*t*_*a*_: Adhesive layer thickness
*A*_*s*_: Cross-sectional area of steel column
*β*: Elastic modulus ratio of CFRP to steel
*A*_*c*_: Cross-sectional area of CFRP
*A*_*0*_: Cross-sectional area of the thick-walled SHS
*ν*_*0*_: Initial bending imperfection value
*L*^*’*^: Effective calculation length
*L*: Total length of the test specimen
*L*_*c*_: CFRP plate length
*L*_*0*_: Length of the thick-walled SHS
*P*_*cr*_: Critical buckling load
*f*_*y*_: Yield stress of steel hollow section
*φ*_*t*_: Stability coefficient of the built-up section
*λ*_*t*_: Equivalent slenderness ratio of the built-up section
$\bar{\lambda }_{{\text{t}}}$: Regularized slenderness ratio of the built-up section
