# Author Correction: Failure mechanism and bearing force of CFRP strengthened square hollow section under compressive load

**DOI:** 10.1038/s41598-024-62442-z

**Published:** 2024-05-27

**Authors:** Can Huang, Yan‑hui Wei, Ke‑jian Ma, Zhuo‑qun Liu, Peng‑gang Tian, Bing‑zhen Zhao

**Affiliations:** 1 Research Center of Space Structures, Guizhou University, Guiyang, 550025 China; 2 Key Laboratory for Structural Engineering of Guizhou Province, Guizhou University, Guiyang, 550025 China; 3 Future City Innovation Technology Co., Ltd., Shaanxi Construction Engineering Holding Group, Xian, 710000 China

Correction to: *Scientific Reports* 10.1038/s41598-024-59752-7, published online 23 April 2024

The Funding section in the original version of this Article was omitted. The Funding section now reads:

“This work was supported by the Science Foundation for Youths of Education Commission of Guizhou Province [2022]109; Guizhou Provincial Program on Commercialization of Scientific and Technological Achievements (No. QKHCG[2023] YB081); Guizhou Provincial Basic Research Program (Natural Science) (No. QKHJC-ZK [2024] YB064); Project of Youth Scholars of Guizhou University [2022]52.”

The original Article has been corrected.

